# AGLR is a novel index for the prognosis of hepatocellular carcinoma patients: a retrospective study

**DOI:** 10.1186/s12893-020-01037-7

**Published:** 2021-02-03

**Authors:** Yan Liao, Rongyu Wei, Renzhi Yao, Liling Qin, Jun Li, Junxiong Yu, Weijia Liao

**Affiliations:** 1 Laboratory of Hepatobiliary and Pancreatic Surgery, Affiliated Hospital of Guilin Medical University, Guilin, 541001 Guangxi People’s Republic of China; 2 Disease Prevention and Control Center of Guilin, Guilin, Guangxi People’s Republic of China; 3 Department of Anesthesiology, The Second Affiliated Hospital of Guilin Medical University, Guilin, 541001 Guangxi People’s Republic of China

**Keywords:** Hepatocellular carcinoma, AGLR, Prognosis, Biomarker

## Abstract

**Background:**

Most hepatocellular carcinoma (HCC) patients’ liver function indexes are abnormal. We aimed to investigate the relationship between (alkaline phosphatase + gamma-glutamyl transpeptidase)/lymphocyte ratio (AGLR) and the progression as well as the prognosis of HCC.

**Methods:**

A total of 495 HCC patients undergoing radical hepatectomy were retrospectively analyzed. We randomly divided these patients into the training cohort (n = 248) and the validation cohort (n = 247). In the training cohort, receiver operating characteristic (ROC) curve was used to determine the optimal cut-off value of AGLR for predicting postoperative survival of HCC patients, and the predictive value of AGLR was evaluated by concordance index (C-index). Further analysis of clinical and biochemical data of patients and the correlation analysis between AGLR and other clinicopathological factors were finished. Univariate and multivariate analyses were performed to identify prognostic factors for HCC patients. Survival curves were analyzed using the Kaplan–Meier method.

**Results:**

According to the ROC curve analysis, the optimal predictive cut-off value of AGLR was 90. The C-index of AGLR was 0.637 in the training cohort and 0.654 in the validation cohort, respectively. Based on this value, the HCC patients were divided into the low-AGLR group (AGLR ≤ 90) and the high-AGLR group (AGLR > 90). Preoperative AGLR level was positively correlated with alpha-fetoprotein (AFP), tumor size, tumor-node-metastasis (TNM) stage, and microvascular invasion (MVI) (all *p* < 0.05). In the training and validation cohorts, patients with AGLR > 90 had significantly shorter OS than patients with AGLR ≤ 90 (*p* < 0.001). Univariate and multivariate analyses of the training cohort (HR, 1.79; 95% CI 1.21–2.69; *p* < 0.001) and validation cohort (HR, 1.82; 95% CI 1.35–2.57; *p* < 0.001) had identified AGLR as an independent prognostic factor. A new prognostic scoring model was established based on the independent predictors determined in multivariate analysis.

**Conclusions:**

The elevated preoperative AGLR level indicated poor prognosis for patients with HCC; the novel prognostic scoring model had favorable predictive capability for postoperative prognosis of HCC patients, which may bring convenience for clinical management.

## Background

Cancer is a significant threat to public health worldwide, and the incidence rate of hepatocellular carcinoma (HCC) has been in a rising trend in recent years [1]. Southeast Asia and sub-Saharan Africa are the high distribution regions of HCC, where chronic hepatitis B virus (HBV) infection is prevalent [2]. Despite the considerable improvement on HCC diagnosis, advancement in surgical resection and liver transplantation in clinical practice, the prognosis of postoperative HCC patients remains unsatisfactory due to the high metastasis and recurrence rates. Therefore, researches on the critical factors affecting prognosis of liver cancer are of great significance to improve the therapeutic efficacy of HCC patients, and promote patient management.

Unlike other cancers, the prognosis of HCC depends not only on tumor malignancy, but also on the remaining liver function. Liver function test is a routine biochemical test used to evaluate liver dysfunction. Alkaline phosphatase (ALP) and gamma-glutamyl transpeptidase (GGT) are the representative enzymes in serum, as well as the parameters for liver function. Previous studies have reported that ALP, GGT and lymphocyte count were independent prognostic predictors for liver cancer [3–5]; and ALP to lymphocyte count ratio or GGT to lymphocyte count ratio could serve as prognostic factors as well [5, 6]. It was found that, the normal references of serum ALP and GGT level were roughly equal in clinical, and a complementary effect was speculated between these two factors; meanwhile, the limitation of a single factor for predicting HCC prognosis should be considered. Therefore, it was assumed that a parameter composed of the two factors may have more favorable prognostic predictive capacity, and a prognosis prediction model made up of multiple factors was constructed: [ALP (U/L) + GGT (U/L)]/lymphocyte count (× 10^9^/L) (AGLR), and this model may have great potential for postoperative prognosis prediction for HCC patients.

## Methods

### Patients

495 HCC patients undergoing surgical resection at the Affiliated Hospital of Guilin Medical University (Guilin, People's Republic of China) from February 2005 to December 2012 conformed to the inclusion criteria of this study. The pathologic examination of HCC was implemented based on the Primary Liver Cancer Clinical Diagnosis and Staging Criteria (Ministry of Health, Beijing, China). The baseline information includes: (1) demographics characteristics: age, gender, drinking, etc.; (2) preoperative laboratory tests: hepatitis B surface antigen (HBsAg), alpha-fetoprotein (AFP), aspartate transaminase, alanine aminotransferase, ALP, GGT, etc.; (3) tumor characteristics: combined with liver cirrhosis, the size and the number of tumors, clinical tumor node metastasis (TNM) stage, microvascular invasion (MVI), recurrence after radical resection, etc. Patients who lost contact during follow-up or with incomplete data were excluded. All methods were carried out abode by the Affiliated Hospital of Guilin Medical University’s guidelines and regulations. This study was approved by the research ethics committee of the Affiliated Hospital of Guilin Medical University and complied with the Declaration of Helsinki Principles. Informed consents were obtained from all patients.

Postoperative long-term follow-up included serum AFP level and abdominal ultrasonography every two months and chest radiography every six months in the first 2 years and at 3- and 6-month intervals respectively after that. Patients would undergo computerized tomography or magnetic resonance imaging scan if recurrence was suspected [7]. Overall survival (OS) was defined as the time from the date of surgery to the date of death or the last follow-up. Disease-free survival (DFS) refers to the time from radical resection to recurrence, metastasis, death or the last follow-up.

### Statistical analysis

All statistical analyses were performed using SPSS 18.0 (SPSS Inc, Chicago, IL) and R (version 3.6.1, R Foundation for Statistical Computing, Vienna, Austria. URL https://www.R-project.org/). The receiver operating characteristic (ROC) curve was used to analyze and calculate the area under the curve (AUC), and the optimal cut-off value was determined by calculating the largest Youden index (sensitivity + specificity—1). Concordance index was determined to predict probability that predicted results were in accordance with the actual results, and C-index greater than 0.5 suggested a certain predictive value of this model. Continuous variables conforming to the normal distribution were expressed as mean ± standard deviation (SD). The comparison of categorical variables was evaluated using the Chi-square test. Kaplan–Meier statistics and Log rank test were used to analyze the different clinical factors related to survival. According to the Cox proportional hazard model, multivariate analysis was performed by SPSS 18.0 (SPSS Inc, Chicago, IL) to explore the independent prognostic value of variables with significance in univariate analysis, and the hazard ratio (HR) and 95% confidence interval (95% CI) were calculated. The survival curves were performed using the Kaplan–Meier method, and the statistical difference of survival distributions between different groups was compared using the log-rank test. Statistical significance was considered if *p* < 0.05.

## Results

### Clinical and biochemical data

We recruited 495 HCC patients and randomly divided them into the training cohort (248 patients) and the validation cohort (247 patients). The mean postoperative follow-up time was 51.6 months (median, 46.0 months; range, 2.0 to 120.0 months). In the training and validation cohorts, the median age of patients was 49.33 and 50.96 years, respectively. The proportion of male patients was much higher than that of female patients, and there were 219 male cases (88.3%) in the training cohort and 213 male cases (86.2%) in the validation cohort, which may be caused by the higher proportion of male liver cancer patients in Asian countries. Clinical and biochemical data were further statistically compared between the training and validation cohorts. The results were shown in Table 1.

**Table 1:** Clinical and biochemical data of examined patients

Parameter	Training cohort	Validation cohort	*p* value
	(n = 248)	(n = 247)	
Basic information			
Age (years)	49.33 ± 11.35	50.96 ± 11.80	0.119
Gender: n (%)			
Female	29 (11.7)	34 (13.8)	0.489
Male	219 (88.3)	213 (86.2)	
Family history: n (%)			
No	216 (87.0)	219 (88.7)	0.435
Yes	32 (13.0)	28 (11.3)	
Drinking: n (%)			
No	140 (56.5)	133 (53.8)	0.560
Yes	108 (43.5)	114 (46.2)	
Smoking: n (%)			
No	148 (59.7)	152 (61.5)	0.577
Yes	100 (40.3)	95 (38.5)	
HBsAg: n (%)			
Negative	41 (16.5)	34 (13.8)	0.391
Positive	207 (83.5)	213 (86.2)	
Minimally invasive surgery: n (%)			
No	184 (74.2)	180 (72.9)	0.739
Yes	64 (25.8)	67 (27.1)	
Lab check data			
WBC (× 10^9^/L)	6.04 ± 2.01	6.39 ± 2.20	0.061
NEUT (× 10^9^/L)	3.65 ± 1.71	3.92 ± 1.73	0.081
LYMPH (× 10^9^/L)	1.64 ± 0.57	1.73 ± 0.65	0.105
Platelets (× 10^9^/L)	173.96 ± 75.18	181.23 ± 79.27	0.096
Albumin (g/L)	39.07 ± 4.59	39.65 ± 4.66	0.220
Globulin (g/L)	31.01 ± 5.80	30.35 ± 6.18	0.215
TBIL (μmol/L)	15.91 ± 14.33	16.45 ± 16.07	0.747
DBIL (μmol/L)	6.33 ± 12.38	6.92 ± 13.17	0.689
ALT (U/L)	45.12 ± 42.93	51.08 ± 46.33	0.783
AST (U/L)	49.98 ± 48.83	51.91 ± 57.49	0.697
ALP (U/L)	95.69 ± 65.26	92.29 ± 42.60	0.493
GGT (U/L): median, range	67.62, 10.7–335.1	72.19, 10.0–351.76	0.854
AGLR level: median, range	90.63, 19.43–441.72	88.83, 16.07–462.16	0.521
AFP (ng/mL): median, range	246.7, 0.20–32,800	220.7, 0.60–25,410	0.363
Pathological features			
Cirrhosis: n (%)			
No	24 (10.0)	13 (5.3)	0.062
Yes	224 (90.0)	234 (94.7)	
Tumor size (cm)	7.81 ± 4.68	7.12 ± 4.11	0.085
Tumor number: n (%)			
Single	190 (76.6)	188 (76.1)	0.896
Multiple	58 (23.4)	59 (23.9)	
TNM stage: n (%)			
I-II	136 (54.8)	124 (50.2)	0.302
III-IV	112 (45.2)	123 (49.8)	
MVI: n (%)			
No	201 (81.0)	188 (76.1)	0.181
Yes	47 (19.0)	59 (23.9)	
Recurrence: n (%)			
No	158 (63.7)	148 (59.9)	0.385
Yes	90 (36.3)	99 (40.1)	

N, number of patients; HBsAg, hepatitis B surface antigen; WBC, white blood cell; LYMPH, lymphocyte count; TBIL, total bilirubin; DBIL, direct bilirubin; ALT, alanine aminotransferase; AST, aspartate aminotransferase; ALP, alkaline phosphatase; GGT, gamma-glutamyl transpeptidase; AGLR, ALP plus GGT to LYMPH; AFP, alpha-fetoprotein; TNM, tumor-node-metastasis; MVI, microvascular invasion

### The relationship between preoperative AGLR level and clinical pathologic characteristics in patients with HCC

**Fig. 1:**
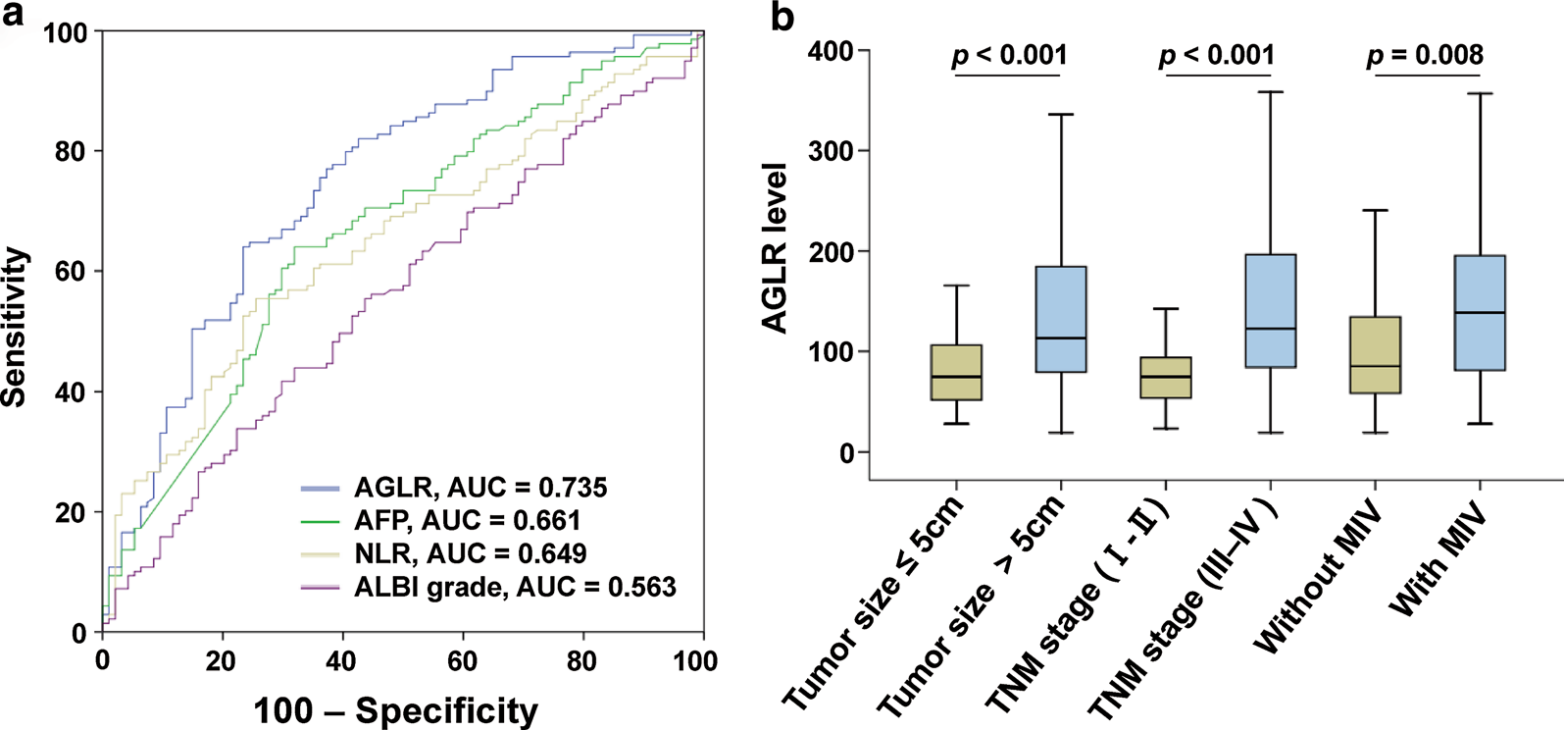
AGLR's predictive capability and its comparison with AFP, NLR and ALBI grade. ROC of AGLR in the training cohort (**a**) and the relationships between AGLR level and tumor size, TNM stage and MVI in the training cohort (**b**)

**Fig. 2:**
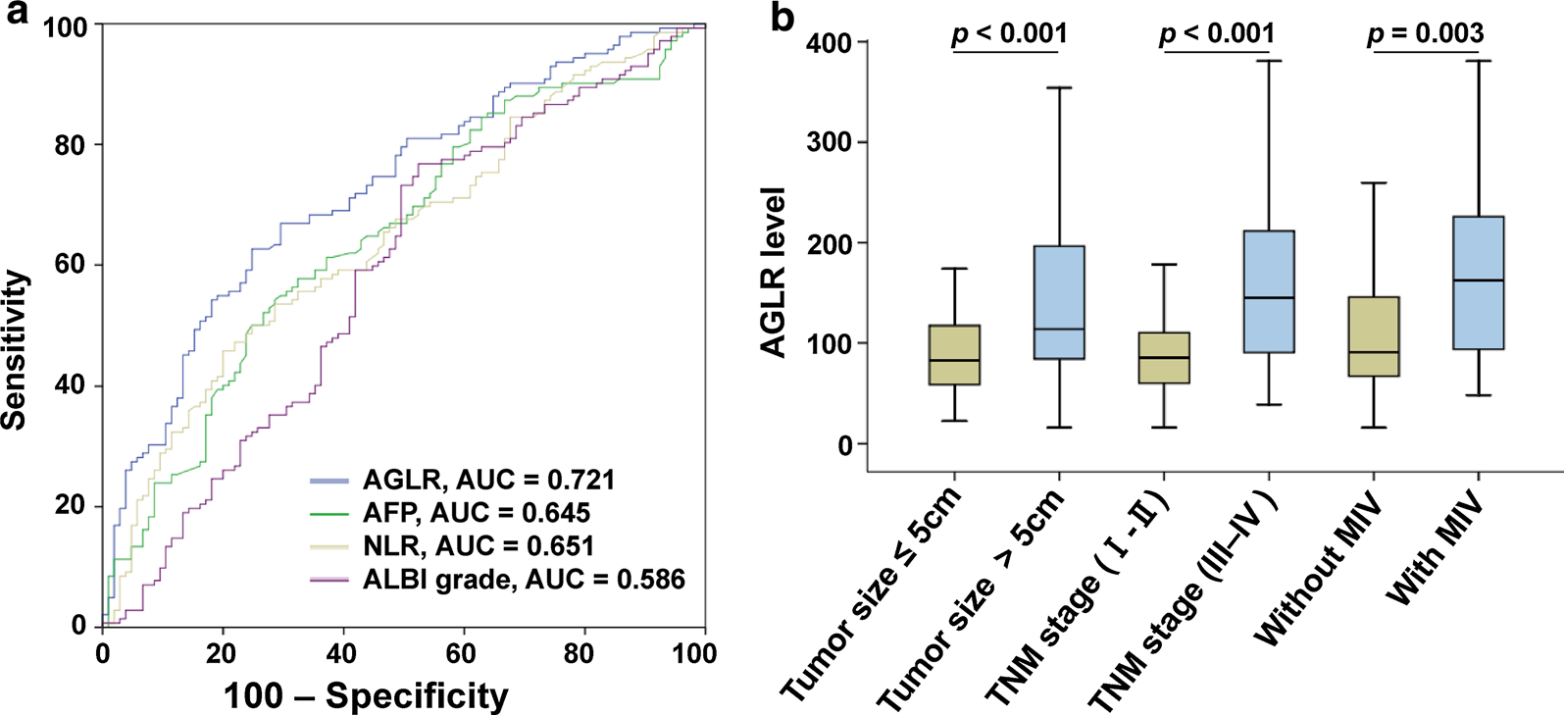
ROC of AGLR in the validation cohort (**a**) and the relationships between AGLR level and tumor size, TNM stage and MVI in the validation cohort (**b**)

Using the receiver operator characteristics (ROC) analysis, the optimal predictive cut-off value of AGLR was 90, with the sensitivity of 75.1%, the specificity of 64.8% and the area under the curve (AUC) was 0.735 (95% CI 0.679–0.786), according to the postoperative survival of HCC patients in the training cohort. Based on this cut-off value, our patients could be divided into two groups by dichotomy: AGLR ≤ 90 and AGLR > 90 groups. Given that serum AFP level is a prognostic factor of liver cancer, and neutrophil-to-lymphocyte ratio (NLR) and albumin bilirubin (ALBI) grade are also common indicators for monitoring the prognosis of liver cancer, we used the same method to calculate the AUCs of AFP, NLR and ALBI, respectively. Interestingly, it was revealed that the AUCs of AGLR were higher than that of AFP, NLR and ALBI in both training cohort and validation cohort (Figs. 1a, 2a). Meanwhile, C-index of AGLR suggested that both AGLR (C-index = 0.637, 95% CI 0.597–0.684) and AFP (C-index = 0.624, 95% CI 0.585–0.671) had predictive value in the training cohort, and more importantly, AGLR had a higher accuracy than AFP; and the value of AGLR (C-index = 0.654, 95% CI 0.613–0.707) and AFP (C-index = 0.577, 95% CI 0.532–0.633) were both verified in the validation cohort. The relationships between preoperative AGLR level and clinicopathologic characteristics were investigated and results were shown in Table 2. In the training cohort (248 patients), high preoperative AGLR level was positively correlated with serum AFP level (> 20 ng/ml) (*p* < 0.001), tumor size > 5 cm (*p* < 0.001), multiple tumors (*p* = 0.035), TNM stage III-IV (*p* < 0.001), presence of MVI (*p* < 0.001). And in the validation cohort (247 patients), high preoperative AGLR level was positively correlated with serum AFP level (> 20 ng/ml) (*p* < 0.001), tumor size > 5 cm (*p* < 0.001), TNM stage III-IV (*p* < 0.001), presence of MVI (*p* < 0.001). However, there were no obvious correlations between AGLR > 90 and age, gender, drinking, HBsAg, liver cirrhosis and recurrence (all *p* > 0.05). Moreover, higher AGLR level was found in tumor size > 5 cm, TNM stage III-IV and MVI patients (*p* < 0.05, Figs. 1b, 2b).

**Table 2:** Correlation between clinical pathologic characteristics and AGLR level in HCC patients

Variables	AGLR level					
	Training cohort (n = 248)			Validation cohort (n = 247)		
	≤ 90 n (%)	> 90 n (%)	*p* value	≤ 90 n (%)	> 90 n (%)	*p* value
Age (years)						
≤ 60	92 (46.2)	107 (53.8)	0.347	81 (41.3)	115 (58.7)	0.815
> 60	19 (38.8)	30 (61.2)		22 (43.1)	29 (56.9)	
Gender						
Female	18 (60.0)	12 (40.0)	0.073	18 (52.9)	16 (47.1)	0.152
Male	93 (42.7)	125 (57.3)		85 (39.9)	128 (60.1)	
Drinking						
No	62 (44.6)	77 (55.4)	0.958	58 (43.3)	76 (56.7)	0.583
Yes	49 (45.0)	60 (55.0)		45 (39.8)	68 (60.2)	
HBsAg						
Negative	14 (38.9)	22 (61.1)	0.444	17 (43.6)	22 (56.4)	0.794
Positive	97 (45.8)	115 (54.2)		86 (41.3)	122 (58.7)	
AFP (ng/mL)						
≤ 20	54 (62.8)	32 (37.2)	** < 0.001**	43 (58.6)	30 (41.4)	** < 0.001**
> 20	57 (35.2)	105 (64.8)		60 (34.5)	114 (65.5)	
Liver cirrhosis						
No	8 (57.1)	6 (42.9)	0.337	10 (43.5)	13 (56.5)	0.856
Yes	103 (44.0)	131 (56.0)		93 (41.5)	131 (58.5)	
Tumor size (cm)						
≤ 5	66 (65.3)	35 (34.7)	** < 0.001**	61 (56.5)	47 (43.5)	** < 0.001**
> 5	45 (30.6)	102 (69.4)		42 (30.2)	97 (69.8)	
Tumor number						
Single	90 (48.6)	95 (51.4)	**0.035**	84 (43.5)	109 (56.5)	0.272
Multiple	21 (33.3)	42 (66.7)		19 (35.2)	35 (64.8)	
TNM stage						
I-II	82 (68.9)	37 (31.1)	** < 0.001**	81 (57.4)	60 (42.6)	** < 0.001**
III- IV	29 (22.5)	100 (77.5)		22 (20.8)	84 (79.2)	
Microvascular invasion						
No	95 (52.2)	87 (47.8)	** < 0.001**	96 (46.2)	112 (53.8)	**0.001**
Yes	16 (24.2)	50 (75.8)		7 (17.9)	32 (82.1)	
Recurrence						
No	70 (47.0)	79 (53.0)	0.388	71 (45.2)	86 (54.8)	0.138
Yes	41 (41.4)	58 (58.6)		32 (35.6)	58 (64.4)	

AGLR, alkaline phosphatase plus gamma-glutamyl transpeptidase to lymphocyte ratio; HBsAg, hepatitis B surface antigen; AFP, alpha-fetoprotein; TNM, tumor-node-metastasis

## Survival analysis based on different preoperative AGLR levels

**Fig. 3:**
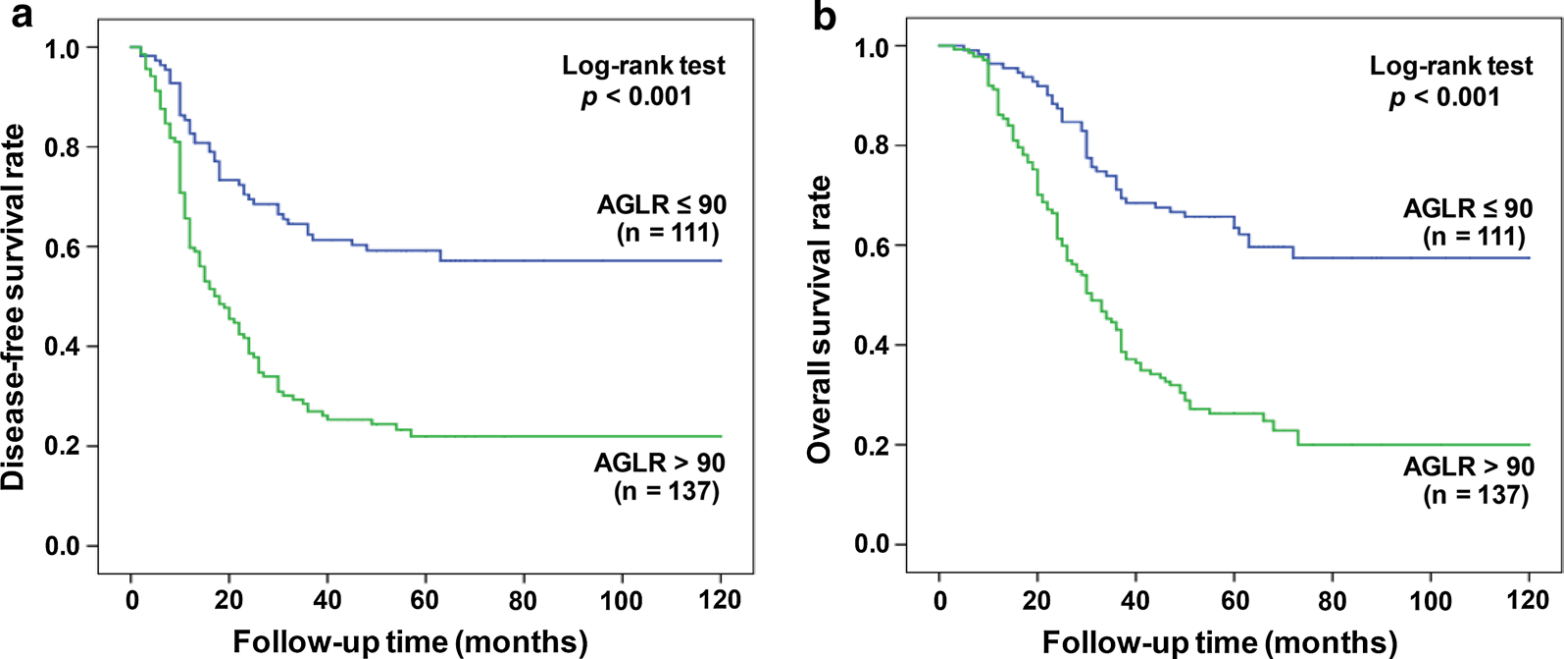
Prognostic significance of AGLR in patients with HCC. Kaplan–Meier analysis of survival in the training cohort. High AGLR level was closely associated with a worse prognosis. The green line represents AGLR level > 90, whereas the blue line represents AGLR level ≤ 90. Kaplan–Meier curves depict OS (**a**) and DFS (**b**) in HCC patients with AGLR > 90 or ≤ 90

In the training cohort, the average survival time for DFS patients with AGLR ≤ 90 was 77.42 months (95% CI 67.70–87.13), and for DFS patients with AGLR > 90, the average survival time was 39.52 months (95% CI 31.90–47.15) (*p* < 0.001, Fig. 3a). Among OS patients, the average survival time of patients with AGLR ≤ 90 was 83.60 months (95% CI 75.18–92.03) and the 1-, 3- and 5-year survival rates were 86.3%, 71.6% and 63.1%, respectively; while for AGLR > 90 patients, they had an average OS of 47.39 months (95% CI 40.26–54.53) and the 1-, 3- and 5-year survival rates were 77.6%, 44.8% and 27.4%, respectively (*p* < 0.001, Fig. 3b).

**Fig. 4:**
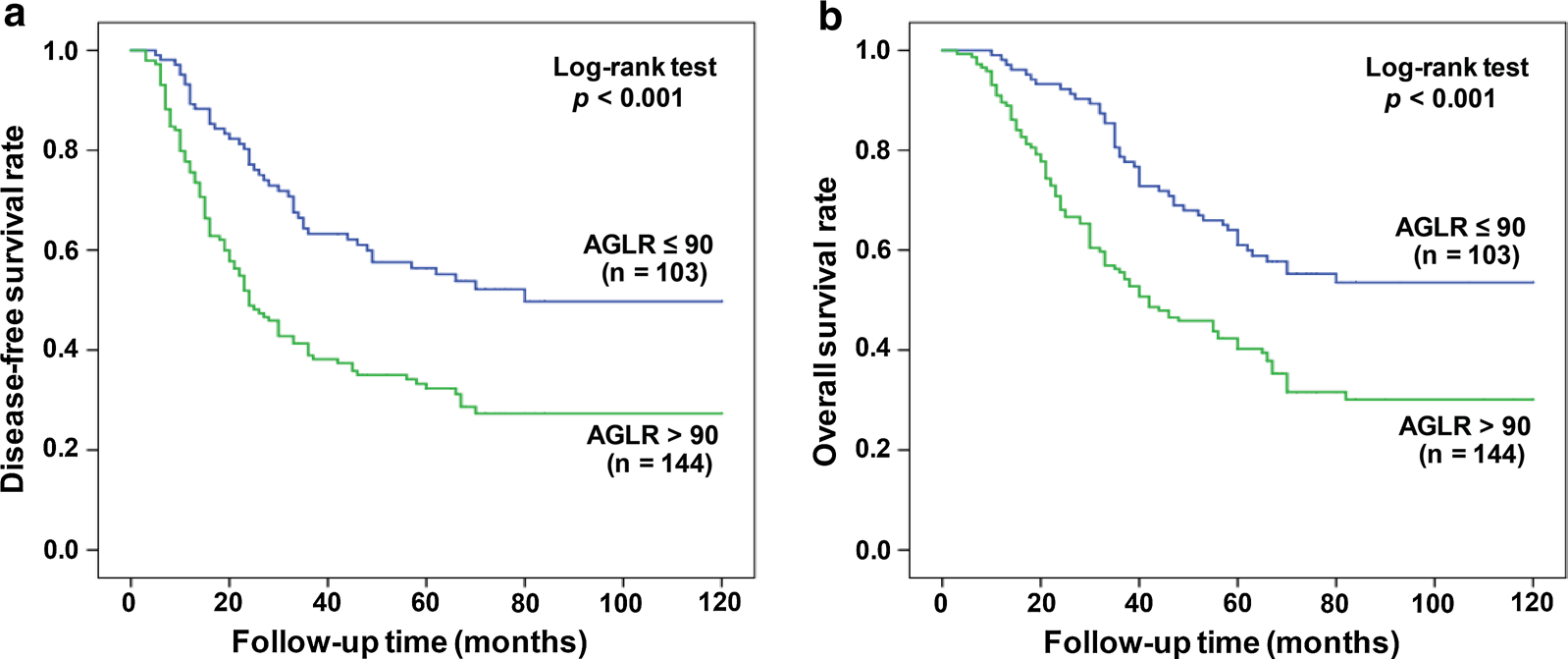
Kaplan–Meier analysis of HCC patients’ survival in the validation cohort. High AGLR level was closely associated with a worse prognosis. Kaplan–Meier curves depict OS (**a**) and DFS (**b**) in HCC patients with AGLR > 90 or ≤ 90

In the validation cohort, the average survival time for DFS patients with AGLR ≤ 90 was 75.58 months (95% CI 66.12–85.03), and for patients with AGLR > 90, the average survival time was 49.28 months (95% CI 41.42–57.14) (*p* < 0.001; Fig. 4a). In OS patients, for HCC patients with AGLR ≤ 90, the average survival time was 83.66 months (95% CI 75.65–91.66) and the 1-, 3- and 5-year survival rates were 88.7%, 73.0% and 60.8%, respectively; and for patients whose AGLR > 90, they had a mean OS of 59.30 months (95% CI 52.10–66.50) and the 1-, 3- and 5-year survival rates were 73.5%, 46.9% and 36.1%, respectively (*p* < 0.001, Fig. 4b).

### Prognostic factors of survival for patients with HCC

The Cox univariate and multivariate regression analyses were applied to evaluate the prognostic value of AGLR and other factors. In the training cohort, it was found that AGLR > 90 (HR = 1.79, 95% CI 1.21–2.69, *p* < 0.001), tumor size (HR = 1.91, 95% CI 1.27–2.61, *p* < 0.001), TNM stage (HR = 1.52, 95% CI 1.03–2.31, *p* = 0.025), MVI (HR = 1.61, 95% CI 1.23–2.39, *p* = 0.007) and recurrence (HR = 2.01, 95% CI 1.47–2.83, *p* < 0.001) were five crucial independent predictors of OS for HCC patients (Table 3), and similar result was found in the validation cohort either (Table 4).

**Table 3:** Univariate and multivariate analysis of overall survival (training cohort, n = 248)

Clinical character	Univariate analysis		Multivariate analysis	
	HR (95% CI)	*p* value	HR (95% CI)	*p* value
AGLR level (> 90 *vs* ≤ 90)	2.66 (2.01–3.88)	** < 0.001**	1.79 (1.21–2.69)	** < 0.001**
Age, years (> 60 *vs* ≤ 60)	1.24 (0.81–1.83)	0.308		
Gender (male *vs* female)	1.23 (0.72–1.91)	0.441		
Drinking (yes *vs* no)	1.03 (0.74–1.41)	0.862		
HBsAg (positive *vs* negative)	1.29 (0.78–2.06)	0.303		
AFP, ng/ml (> 20 *vs* ≤ 20)	1.71 (1.19–2.47)	**0.003**	1.13 (0.77–1.67)	0.514
Liver cirrhosis (yes *vs* no)	1.03 (0.51–2.09)	0.930		
Tumor size, cm (> 5 *vs* ≤ 5)	2.86 (1.97–3.91)	** < 0.001**	1.91 (1.27–2.61)	** < 0.001**
Tumor number (multiple *vs* single)	1.60 (1.13–2.26)	**0.006**	1.12 (0.81–1.53)	0.460
TNM stage (III–IV *vs* I–II)	1.96 (1.39–2.77)	** < 0.001**	1.52 (1.03–2.31)	**0.025**
MVI (yes *vs* no)	2.57 (1.93–3.74)	** < 0.001**	1.61 (1.23–2.39)	**0.007**
Recurrence (yes *vs* no)	2.70 (1.69–3.59)	** < 0.001**	2.01 (1.47–2.83)	** < 0.001**

CI, confidence interval; HR, hazard ratio; AGLR, alkaline phosphatase plus gamma-glutamyl transpeptidase to lymphocyte ratio; HBsAg, hepatitis B surface antigen; AFP, alpha-fetoprotein; TNM, tumor-node-metastasis; MVI, microvascular invasion

**Table 4:** Univariate and multivariate analysis of overall survival (validation cohort, n = 247)

Clinical character	Univariate analysis		Multivariate analysis	
	HR (95% CI)	*p* value	HR (95% CI)	*p* value
AGLR level (> 90 *vs* ≤ 90)	2.47 (1.59–3.64)	** < 0.001**	1.82 (1.35–2.57)	** < 0.001**
Age, years (> 60 *vs* ≤ 60)	1.16 (0.77–1.76)	0.460		
Gender (male *vs* female)	1.49 (1.11–2.30)	0.096		
Drinking (yes *vs* no)	1.23 (0.88–1.71)	0.207		
HBsAg (positive *vs* negative)	1.10 (0.72–1.67)	0.639		
AFP, ng/ml (> 20 *vs* ≤ 20)	1.65 (1.13–2.65)	**0.011**	1.07 (0.81–1.44)	0.366
Liver cirrhosis (yes *vs* no)	1.03 (0.59–1.79)	0.801		
Tumor size, cm (> 5 *vs* ≤ 5)	2.11 (1.49–2.91)	** < 0.001**	1.69 (1.15–2.480)	**0.008**
Tumor number (multiple *vs* single)	1.63 (1.22–2.54)	**0.013**	1.20 (0.81–1.79)	0.307
TNM stage (III–IV *vs* I–II)	2.33 (1.83–3.50)	** < 0.001**	1.39 (1.07–2.10)	**0.016**
MVI (yes *vs* no)	2.81 (1.99–3.73)	** < 0.001**	2.30 (1.57–2.93)	** < 0.001**
Recurrence (yes *vs* no)	2.51 (1.64–3.51)	** < 0.001**	2.05 (1.46–2.79)	** < 0.001**

CI, confidence interval; HR, hazard ratio; AGLR, alkaline phosphatase plus gamma-glutamyl transpeptidase to lymphocyte ratio; HBsAg, hepatitis B surface antigen; AFP, alpha-fetoprotein; TNM, tumor-node-metastasis; MVI, microvascular invasion

**Fig. 5:**
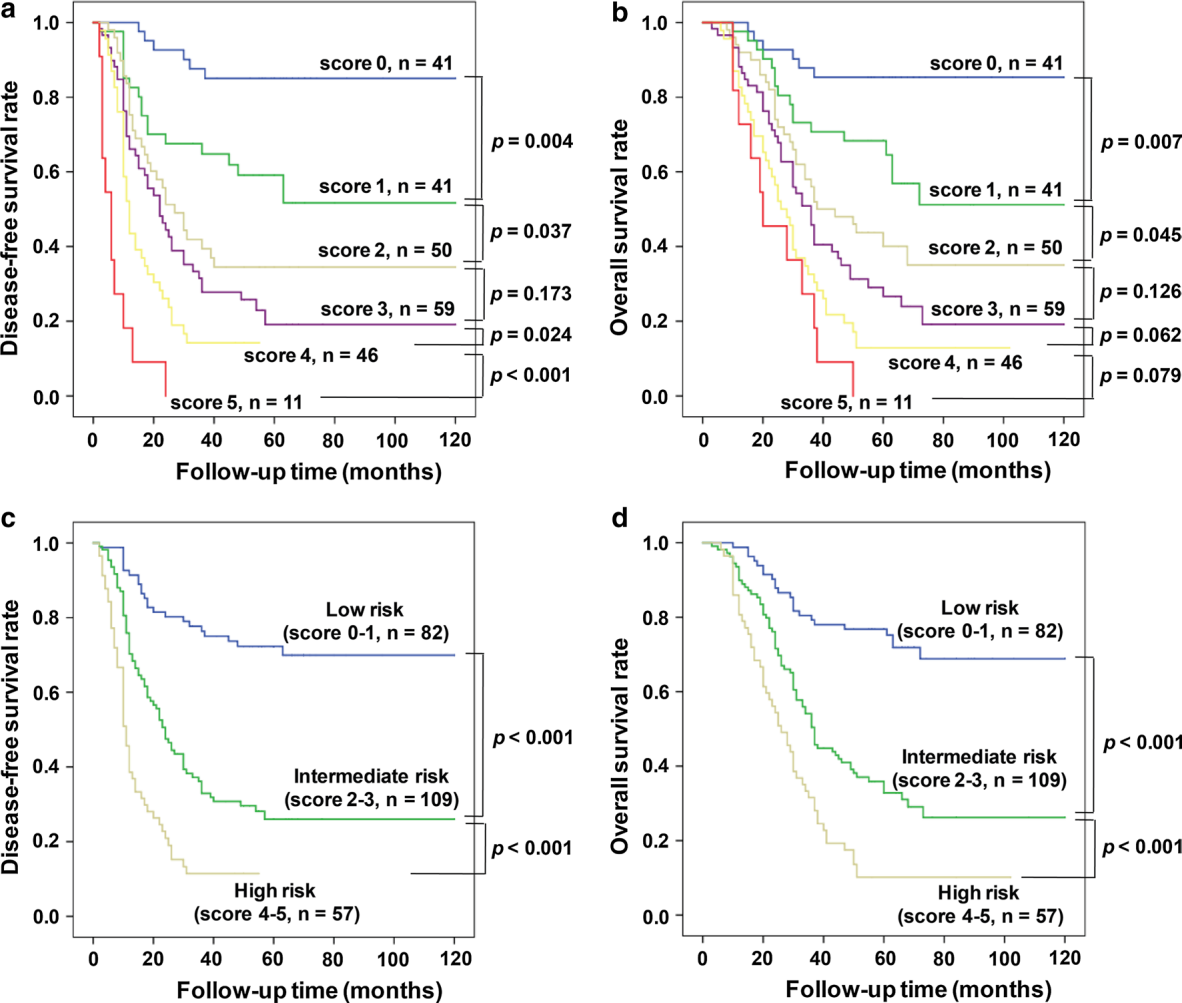
In the training cohort, comparison of prognostic effects of different scoring groups, there was no statistical significance of survival between patients with a score of 2 *vs.* 3, for both DFS (**a**) (*p* = 0.173) and OS (**b**) (*p* = 0.126). Kaplan–Meier analysis of survival for different risks groups. There were statistical significance between the low-, medium- and high-risk groups (all *p* < 0.001) for both DFS (**c**) and OS (**d**)

**Fig. 6:**
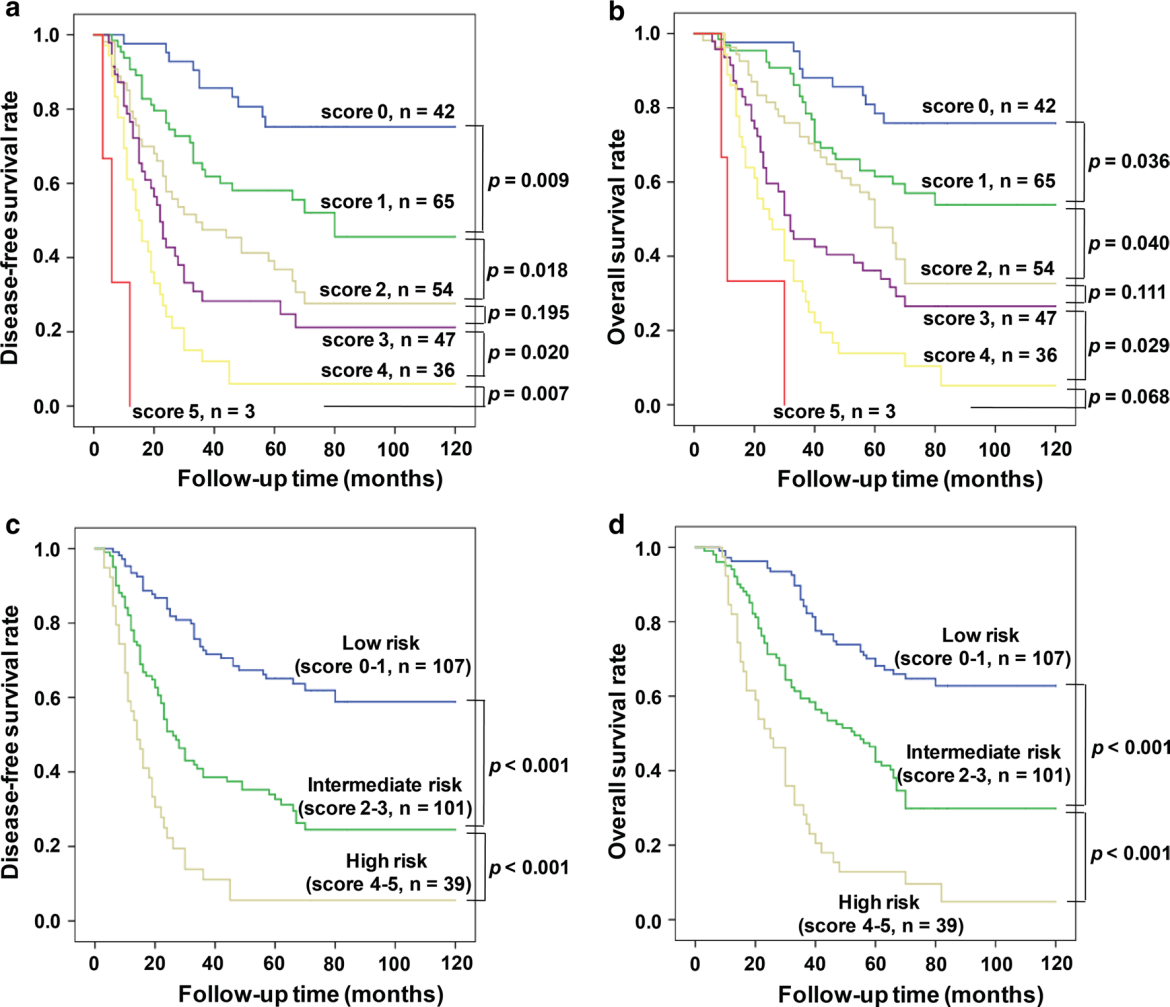
In the validation cohort, DFS’s patients with a score of 2 *vs.* 3 (*p* = 0.195) (**a**), OS’s patients with a score of 2 *vs.* 3 (*p* = 0.111) and 4 *vs.* 5 (*p* = 0.068) (**b**) had no statistical significance. There were statistical significance between the low-, medium- and high-risk groups (all *p* < 0.001) for both DFS (**c**) and OS (**d**)

Then, each of the above five independent predictors were assigned, such as AGLR ≤ 90 was assigned 0 point and AGLR > 90 was assigned 1 point, and other four predictors were assigned in the same manner. Thus, all HCC patients would be divided into six groups of different scores, ranging from 0 to 5 points, based on their accumulated total scores. As the result, a new prognostic scoring model consisted of multiple variables was constructed. However, some comparisons between two of these new groups had no statistical different. For instance, in the training cohort, for DFS patients with a score of 2 vs*.* 3 (*p* = 0.173) (Fig. 5a) and for OS patients with a score of 2 vs. 3 (*p* = 0.126), score 3 vs. 4 (*p* = 0.062) and score 4 vs. 5 (*p* = 0.079) (Fig. 5b). Similar result was also found in the validation cohort (Fig. 6a, b). In view of these circumstances and in order to obtain better application value of this new model, we further divided these groups according to the scores: 0–1 points (low-risk group), 2–3 points (medium-risk group) and 4–5 points (high-risk group). Surprisingly, the survival analyses revealed that in both training cohort (Fig. 5c, d) and validation cohort (Fig. 6c, d), HCC patients’ postoperative survival time had significant differences between the low-, medium- and high-risk groups, which was an obviously decreasing trend.

## Discussion

In this study, we established a simple and evidence-based prognostic model, named AGLR, in order to predict the risk of survival for HCC patients undergoing radical resection, which incorporated routinely available laboratory parameters: ALP, serum GGT level and lymphocyte count. And this prognostic model was repeatable and accurate. Several studies have shown that elevated serum ALP level may be related to some pathological conditions [8–10], and other studies have revealed that ALP was a cancer-associated serum enzyme [11–13]. Moreover, according to the electron microscopic cytochemistry, ALP was observed to contain the nuclear localization signal and was linked to the proliferation of cancer cells [14]. Therefore, the elevation of serum ALP may play an essential role in cancer proliferation. GGT is an ubiquitous epithelial enzyme that associated with higher mortality in many diseases, including liver disease, pancreatic disease, renal failure, myocardial infarction and diabetes [15]. Lymphocytes may play an important role in immune regulation of tumor; T cells could be activated by phytohemagglutinin (PHA), ionomycin (Iono) and other factors; in the meanwhile, T cells could be induced to apoptosis through a variety of ways [16]. Moreover, some researches revealed that reduced CD8^+^ T lymphocytes might have relation to unfavorable prognosis of liver cancer [17, 18]. Therefore, all the three factors mentioned above are adverse factors for HCC patients; if all of them can be taken into consideration when predicting liver cancer patients’ postoperative prognosis, more reliable prediction and preciser medical treatment will realize.

In this retrospective study, we first analyzed the clinical and biochemical data of training cohort and validation cohort, as well as the relationship between preoperative AGLR level and clinical characteristics of patients with HCC. It is noteworthy that elevated preoperative AGLR level is positively related to tumor size > 5 cm, TNM stage III-IV and MVI. This result was further confirmed in the validation cohort. However, MVI, as a unique way for HCC cells to invade the blood vessel, depends on the invasive and metastasizing potential of liver cancer cells. Therefore, it was speculated that elevated AGLR level may endow cancer cells with the possibility of invasion and metastasis through changing its micro-environment for metabolism, and further lead to the deterioration of HCC.

In addition to AGLR, AFP, tumor size, TNM stage, MVI and recurrence were also associated with a shorter OS for HCC patients. Previous studies have found that AFP promoted the invasion and metastasis of HCC cells by up-regulating the expression of metastasis related proteins [5], therefore, AFP was an unfavorable prognostic predictor. Tumor size is an important prognostic marker for HCC [3, 19]. Patients with a single tumor > 5 cm or multiple tumors would have a higher probability of bilobar involvement, invasion of microvascular and adjacent organs as well as the histologically positive margins [20]. MVI, which can lead to early postoperative recurrence and metastasis, is a significant risk factor of poor prognosis for HCC patients after radical resection as well as an independent predictor of long-term postoperative survival [21].

In recent years, many molecular biological models of liver cancer have been reported [22–24]. NLR and ALBI grades are simple and easy to determine preoperative predictors. In this study, we also analyzed the predictive effects of these two scoring models on the prognosis of liver cancer. The results are consistent with some previous studies [25–28], NLR and ALBI grade have predictive significance in HCC patients. Interestingly, we found that the predictive ability of AGLR was better than that of NLR and ALBI. Since the change of albumin level is related to the prognosis of HCC, Shen et al. proposed that albumin to gamma-glutamyltransferase ratio (AGR) level might be related to the prognosis of HCC, and AGR classification was compared with platelet-to-lymphocyte ratio (PLR), it was found that the AGR-PLR score can further stratify HCC patients with different prognosis, and has a stronger predictive ability [29]. In this study, AGLR has the characteristics of simplicity and low cost, and it was confirmed that high levels of AGLR are associated with poor prognosis of HCC. In addition, we also found that AGLR level (> 90), tumor size > 5 cm, TNM stage III-IV, presence of MVI and recurrence were independent predictors of HCC survival by the multivariate analyses in both training and validation cohort. Considering the heterogeneity of prognosis, the predictive value of a single factor has certain of limitations, we established a simple prognostic scoring model based on the five independent predictors, which can be readily available in daily practice. All the 495 HCC patients were randomly divided into the training cohort and validation cohort, and patients were further separated into the six different scoring groups. After that, we optimized this scoring model by changing the six scoring groups into the low-, medium- and high-risk groups, which could better predict different risks of survival. This new prognostic scoring model has potential application value in prognosis and can better predict the outcome of HCC patients. In the future, we will also consider comparing and merging AGLR with other prognostic scoring models to further improve the effect of predicting the prognosis of HCC patients and determine appropriate intervention.

There are some limitations in this study yet. Firstly, this is a retrospective study based on limited data of HCC patients from a single hospital, and only HCC patients accepted radical resection were enrolled in this study; thus, AGLR’s prognostic prediction value for patients accepted liver transplant or TACE needs further study. Secondly, eastern and western countries’ opinions on the surgical indications for HCC are still controversial [30], Third, the environmental background of HCC patients from different regions varies with each other. For instance, in China, the proportion of HBV-related HCC is nearly 90%; whereas in western countries, most HCC are caused by alcoholic cirrhosis, non-alcoholic fatty liver disease and HCV infection [31]. Therefore, patients enrolled may suffer from obvious limitations of regional factors. For future researches, multicenter external validations and prospective studies are needed, so as to verify that this novel model may be widely available for HCC patients.

## Conclusions

High preoperative AGLR level predicted poor prognosis for HCC patients; the simple and novel prognostic scoring model could effectively identify the higher risk of poor survival and early recurrence and may help select an appropriate treatment based on different risk stratification.

## Data Availability

The datasets used and/or analysed during the current study are available from the corresponding author on reasonable request.

## Abbreviations

HCC: Hepatocellular carcinoma
HBV: Hepatitis B virus
ALP: Alkaline phosphatase
GGT: Gamma-glutamyl transpeptidase
HBsAg: Hepatitis B surface antigen
AFP: Alpha-fetoprotein
NLR: Neutrophil-to-lymphocyte ratio
ALBI: Albumin bilirubin
AGR: Gamma-glutamyltransferase ratio
PLR: Platelet-to-lymphocyte ratio
TNM: Tumor node metastasis
MVI: Microvascular invasion
DFS: Disease-free survival
OS: Overall survival
CI: Confidence interval
HR: Hazard ratio
ROC: Receiver operating characteristic
VS: Versus
